# Multitechnique Approach for Xylazine Detection: Chemical Spot Tests, Immunoassays, and Scanning Electron Microscopy

**DOI:** 10.1021/acsomega.5c07057

**Published:** 2025-12-03

**Authors:** Katherine Davis, Leon Lippincott, Danielle Malenius, Rida Saleem, Shanlin Fu, Saman Majeed

**Affiliations:** † Department of Chemistry and Biochemistry, 8620 University of North Carolina Asheville, One University Heights, Asheville, North Carolina 28804, United States; ‡ Centre for Forensic Science, University of Technology Sydney, Ultimo, NSW 2007, Australia

## Abstract

The infiltration of xylazine into the illicit drug supply, particularly as a fentanyl adulterant, has raised significant safety concerns due to its severe toxicological effects and the absence of an effective antidote for targeted therapeutic intervention. Despite the growing implications of xylazine in overdose cases, presumptive testing methods for its detection remain underdeveloped in forensic and public health settings. This study addresses the urgent need for reliable screening tools by evaluating the applicability of chemical spot tests, microcrystalline tests, commercially available xylazine immunoassay test strips, and other field-deployable drug test kits. A comprehensive panel of chemical spot test reagents was tested, and cobalt thiocyanate, Eosin Y (pH 7.0), and Liebermann, Mandelin, Mecke, Marquis, Scott, and Young reagents produced distinct and rapid color changes with xylazine. In parallel, different microcrystalline test reagents were evaluated, and the resulting microcrystal morphology was assessed by using light microscopy and scanning electron microscopy (SEM). Gold chloride reagent yielded immediate, reproducible, and well-defined microcrystals across different xylazine formulations, establishing it as the most effective reagent for microcrystalline testing. All xylazine test strips yielded clear positive test results when tested with xylazine. In contrast, many commercial and on-site screening tools displayed cross-reactivity or false-positive results with xylazine, raising concerns about its specificity and reliability. These findings highlight the need to develop and validate xylazine-specific presumptive screening tests to facilitate forensic investigations, support harm reduction efforts, and enable informed, safer decision-making by people who use drugs (PWUDs).

## Graphical abstract

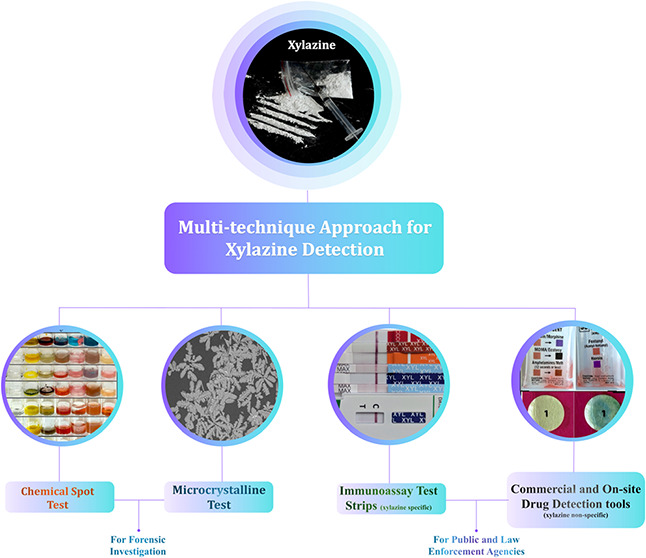

## 1 Introduction

Xylazine, first developed as an antihypertensive agent in the Federal Republic of Germany by Farbenfabriken Bayer in 1962,^1^ has recently emerged as a prevalent adulterant in fentanyl drug samples.^2^ Xylazine is a nonopioid drug and an analogue of clonidine, phenothiazines, and tricyclic antidepressants (TCAs). The chemical name, molecular formula, and relative molecular mass of xylazine are 5,6-dihydro-2-(2,6-xylidino)-4*H*-1,3-thiazine, C_12_H_16_N_2_S, and 220.33, respectively.^3^ The US Food and Drug Administration (FDA) approved its use as an analgesic and sedative exclusively for use in veterinary medicine in 1972.^4^ However, studies in humans were terminated due to severe hypotension and bradycardia, and it was never approved for human use due to its potentially life-threatening adverse effects.^5,6^

In 2001, the US Drug Enforcement Administration (DEA) reported for the first time the illicit human consumption of xylazine in Puerto Rico.^4^ Over the next decade, xylazine-involved overdose cases were observed and steadily increased in the cattle-farming communities across Puerto Rico, most likely due to the diversion of xylazine from veterinary sources to the illicit drug market.^7^ By 2019, xylazine was detected in 1.8% of overdose deaths across 38 U.S. states, and by 2022, it was found in seized drugs in 48 U.S. states.^2,8^ Later, fentanyl adulterated or associated with xylazine (FAAX) was declared an emerging public health threat by the Office of National Drug Control Policy (ONDCP) in April 2023, and the subsequent FAAX report released in June 2024 emphasized the urgent need for public safety measures against xylazine exposure.^9,10^

The infiltration of xylazine into the street drug supply has increased dramatically, as it is either unknowingly consumed as an adulterant or deliberately added by people who use drugs (PWUDs) to prolong the high.^11,12^ Chronic use of xylazine is reported to impact multiple organs, including but not limited to depression, tissue necrosis, skin ulcers, hyperglycemia, osteoporosis, apnea, bradycardia, and euphoria. Xylazine toxicity has been reported to cause dysarthria, disorientation, sedation, hyporeflexia, hypotension, and bradycardia, and its effects may not be reversible with the typical opioid antidote, naloxone.^10,11,13^ Prolonged apnea leading to hypoxic brain injury, respiratory failure, and severely necrotic skin ulcers are reported as complications secondary to xylazine.^5,11,14–16^

The ONDCP reports strongly emphasize the critical importance of the development and standardization of analytical methods and protocols across forensic drug laboratories, public health agencies, and medical examiner offices.^9,10^ Despite the increasing prevalence of xylazine adulteration, only one study has reported xylazine detection using a chemical spot test involving three color test reagents, with a lack of validation and standardization across multiple reagents and other presumptive detection methods.^17^ Other studies primarily utilize immunoassay-based test strips or dipsticks for xylazine screening, in pure drug substances, in addition to biological samples such as urine.^18–20^ However, they are limited by poor suitability for powder samples, cross-reactivity, and potential false positives with structurally similar compounds such as lidocaine and methamphetamine.^17–21^

Chemical spot tests and microcrystalline tests, routinely used in forensic laboratories, have not been systematically evaluated for xylazine detection, creating a gap in reliable, laboratory-based preliminary screening. To address these limitations, this study systematically evaluated the performance characteristics, including reliability, specificity, and operational feasibility, of several screening approaches for xylazine detection. The potential application of chemical spot test, microcrystalline test, immunoassay test strips, and commercially available or field-deployable drug test kits was evaluated to identify reliable options for forensic laboratories and on-site screening of xylazine. By integrating these complementary techniques, this approach aimed to enhance the specificity and sensitivity of xylazine detection in illicit powders and solution-based samples, offering a versatile, quick, and adaptable analytical solution for diverse sample types. The results of this study are intended to advance harm reduction efforts, strengthen forensic investigations, and enable the PWUDs to make safer, informed decisions regarding substance abuse and drugs adulterated with xylazine.

## 2 Experimental Section

### 2.1 Chemicals and Reagents

Chemicals used in this study included xylazine (hydrochloride) purchased from Cayman Chemical Company (MI). The chemicals used for the preparation of chemical spot reagents, including acetaldehyde, ethanol, *meta*-dinitrobenzene, and isopropyl amine, were purchased from Sigma-Aldrich (MO). Ammonium metavanadate, formaldehyde, potassium hydroxide, sodium nitroprusside, sodium carbonate, sodium nitrite, sulfuric acid, cobalt­(II) thiocyanate, methanol, glacial acetic acid, vanillin, hydrochloric acid, chloroform, ferric chloride hexahydrate, molybdic acid, acetic anhydride, and pyridine were purchased from Fisher Scientific (PA). Cobalt­(II) acetate dihydrate and copper­(II) sulfate pentahydrate were purchased from Oakwood Chemical (SC). Selenious acid was purchased from Thermo Scientific (NJ), and Eosin Y was from AmBeed (NJ). Acetone and ultrapure water were purchased from VWR (PA). Microscope slides and chemicals used for the preparation of microcrystalline reagents, including ammonium thiocyanate, mercuric chloride, gold chloride, dihydrogen hexachloroplatinate­(IV) hexahydrate, and silver nitrate, were purchased from Fisher Scientific (PA). Stannous chloride was purchased from Thermo Scientific (NJ).

### 2.2 Chemical Spot Tests

Chemical spot tests allow forensic laboratories, law enforcement, and border security personnel to quickly identify the presence of specific substances. The simplicity and speed of the color test make it attractive to incorporate in research and investigation. The color test reagent chemically reacts with a specific functional group or molecular structure of the analyte/drug, and this reaction yields charged organic species and colored metal complexes, depending on the analyte/drug tested.^22,23^ The color of the reaction mixture is then compared visually with reference charts, the current standard being the Munsell color charts and color identification system illustrated in Clarke’s Analysis of Drugs and Poisons.^24–27^ This system identifies 10 primary colors, which include black, blue, brown, green, gray, orange, pink, red, violet, and yellow, with variations in hue represented by combining 2 colors (e.g., green-blue) where the second color signifies the dominant hue.^22^

The xylazine samples used for chemical spot testing consisted of a solid powder only. The chemical spot test was conducted by labeling spot plates for quality control (QC), negative, and test samples. Subsequently, 3–4 drops of the chemical reagents were added to each designated well. A pinhead-sized amount of xylazine powder (approximately 0.1 × 0.2 mg) was introduced into the reagent in the test well and gently stirred. The plate was left undisturbed at room temperature, and any color transition was carefully observed and documented both prior to and following the addition of xylazine. The colorimetric spot test reagents were prepared following the protocols described by O’Neal et al.,^25^ and color transitions were recorded according to the color identification system elucidated in Clarke’s Analysis of Drugs and Poisons.^26,27^ Each reagent was tested against the xylazine sample in triplicate using the above-mentioned protocol.

### 2.3 Point-of-Care and Field-Deployable Drug Screening Tools

The commercially available options for xylazine testing evaluated in this study included xylazine-specific immunoassay test strips/dipsticks in addition to commercial and on-site drug detection tools. Specifically, xylazine immunoassay test strips and urine dipsticks were assessed to evaluate their reactivity and effectiveness in detecting xylazine. The xylazine test strips were purchased from six different commercial vendors, namely, Rapid Response Xylazine Test Strip from BTNX Inc. (ON, Canada), Xylazine Test Strip Kit from MD-Bio (TX), Xylazine Test Strip from DanceSafe/WHPM Inc. (CA), Xylazine Drug Test Strip from WaiveDx (CA), Xylazine Test Strip from WiseBatch HarmReduction (CA), and Xylazine Drug Test Strip from 12 PanelNow (FL). Additionally, the Xylazine Urine Dip Test was purchased from Medimpex United Inc. (PA). All tests were performed using xylazine solution samples prepared in ultrapure water at a concentration of 1 mg/mL (1000 μg/mL). Each test was conducted following the procedures described in the respective manufacturer’s instruction manuals or user guides, and results were analyzed and documented accordingly. Each test strip or dipstick was unsealed immediately prior to testing and immersed vertically into a xylazine solution for 10–15 s, or as specified by the manufacturer. After immersion, the strip or dipstick was placed horizontally on a clean, nonabsorbent surface. The results were interpreted and photographed after the manufacturer’s prescribed time of application. Generally, the appearance of a single purple band at the control line (C-band) signifies a positive result for xylazine, while the presence of both a test band and a control band (T and C) indicates a negative result.

On-site drug screening tools restricted to law enforcement agencies, such as multireagent field test kits and narcotics field test tubes, were also evaluated for their cross-reactivity with xylazine. These kits mostly encompass one or more chemical reagents analogous to those used for drug detection in forensic laboratories and operate on the same principle as the chemical spot test. On-site field test kits analyzed in this study included a NARKII narcotics analysis reagent kit, in addition to narcotics field test tubes, viz., NARK Mandelin Reagent, NARK Marquis Reagent, NARK Dille-Koppanyi Reagent, and GHB Reagent purchased from Sirchie (NC). Narcotics identification system single-use tests were procured from Nik Public Safety (FL). For the multireagent on-site field-testing kits, including the narcotics analysis reagent kit and narcotics field test tubes, xylazine (solid powder) in pinhead-sized amounts (0.1–0.2 mg) was introduced into the pouch encompassing glass ampules or test tubes. The reagent-containing ampules or test tubes were then broken in sequence (typically from left to right), and the kit or tube was agitated as per the protocol. Color changes were observed and recorded.

Additionally, xylazine was evaluated for cross-reactivity with drug detection tools intended for general public use, including drug detection wristbands, stickers, and wipes. According to the manufacturers’ guidelines, a transition to blue indicates the potential presence of flunitrazepam (“roofie”) with the wristband and GHB with the sticker, while the appearance of pink on the detection wipe is interpreted as a presumptive indication of fentanyl or related opioids. Xylazine was tested with the DrinkCheck Wristband Roofie Test, manufactured by Xantus (DE), and Spiked Drink Tester, G-Check Sticker, manufactured by Philmedi (Kyonggi-do, South Korea), in addition to F-1 fentanyl/opioid detection wipes procured from Trace Eye-D (CA). In the case of the drug detection wristband, 3–4 drops of 1 mg/mL (1000 μg/mL) xylazine test solution were placed onto the test spot, gently rubbed, and left for approximately 10 s, after which the area was observed for a visible color change. However, with drug test stickers, the protective film was peeled away, and a drop of xylazine solution was applied directly to the exposed area. The solution was gently rubbed to ensure complete absorption and then allowed to air-dry thoroughly. Color changes were recorded approximately 1 min after application. For the analysis of drug detection wipes, a pinhead-sized amount (0.1–0.2 mg) of xylazine was deposited onto a clean surface and subsequently wiped using the detection wipe. The wipe was allowed to sit for a few seconds, after which it was examined for any observable color change, indicating a presumptive reaction.

### 2.4 Microcrystalline Test

The microcrystalline test is a reversible and nondestructive analytical technique used for the presumptive identification of drugs, where microcrystals with distinctive morphological characteristics are formed through the reaction of a specific analyte with a reagent. This method allows for detailed observation of the shape, size, color, and spatial arrangement (habit) of the resulting microcrystals, offering enhanced specificity in substance identification. Owing to its high discriminating power, the microcrystalline test can not only distinguish between various drugs but also differentiate between enantiomers and racemic mixtures.^28–30^

The microcrystalline reagents for this study were selected from the reagent list reported by Brinsko et al.^30^ and Quinn et al.^31,32^ including 5% dihydrogen hexachloroplatinate­(IV) hexahydrate (H_2_PtCl_6_·6H_2_O), 0.5% gold chloride (HAuCl_4_·3H_2_O), 5% mercuric chloride (HgCl_2_), 5% silver nitrate (AgNO_3_), and 1% stannous chloride (SnCl_2_·2H_2_O) aqueous solutions.^31^ The xylazine samples used for microcrystalline testing included solid powders as well as solutions prepared in ultrapure water and methanol at a concentration of 1 mg/mL (1000 μg/mL). For the solution samples, a single drop (∼10 μL) of an aqueous or methanolic xylazine solution was applied. For solid samples, a pinhead-sized quantity of xylazine was carefully added to the reagent droplet on the slide. The microcrystalline reagent was dispensed first to record a QC negative, followed by observing xylazine aqueous and methanolic solutions (QC positive). Microcrystalline testing was conducted by combining xylazine with different microcrystalline reagents on a clean microscope slide, with each test performed in triplicate to ensure reproducibility. The drug–reagent mixture was gently mixed on the slide surface to ensure uniformity, and the slides were left undisturbed at room temperature to allow crystal formation. Microcrystal development was monitored under a Nikon Eclipse E200 compound microscope at varying magnifications (4×, 10×, 20×, and 40×). Images were captured using a Moticam A8 camera and processed using Motic Images Plus 3.1 software. The description of typical crystal morphology was referred to the description illustrated in Clarke’s Analysis of Drugs and Poisons and the National Institute of Justice (NIJ)-sponsored project titled “modern compendium of microcrystal tests for illicit drugs and diverted pharmaceuticals”.^27,30,32^

### 2.5 Scanning Electron Microscopy (SEM) Characterization

During standard microcrystalline observation under a light microscope, the surface tension of conventional microscope glass slides plays a critical role in influencing both the morphology and the spatial distribution of crystal growth. These hydrophilic surfaces promote lateral spreading and aggregation of the microcrystal clusters. In contrast, carbon-coated conductive adhesive tapes or carbon stubs, commonly used for scanning electron microscopy (SEM), present hydrophobic surfaces, minimize static charge accumulation, and favor the vertical growth of drug–reagent microcrystals.^28^

Microcrystalline reagents yielding immediate and distinct microcrystals with xylazine under a light microscope were further examined by using SEM for detailed morphological characterization. Drug–reagent samples were prepared on carbon-coated conductive adhesive tape mounted on a sample stub for SEM imaging. All samples were analyzed using JSM-IT700HR SEM (JEOL Ltd., Japan) equipped with a backscattered electron detector (BED-C). Imaging was conducted at an accelerating voltage of 20 kV, with a working distance (WD) of approximately 11 mm, a chamber pressure of 70 Pa, and a probe current of 50 pA. Microcrystals were visualized and documented at three magnification levels: 2, 5, and 10 μm to capture detailed morphological features.

### 2.6 Performance Evaluation of Xylazine Detection Methods

The analytical performance of xylazine detection methods, viz., chemical spot test, immunoassay test, and microcrystalline testing, was evaluated to assess their sensitivity, specificity, and reliability. Chemical spot tests, immunoassay tests, and microcrystalline testing were performed to evaluate all three performance parameters, following the procedures outlined in Sections 2.2, 2.3, and 2.4, respectively. For both chemical spot tests and microcrystalline tests, only reagents yielding distinct and reproducible responses were assessed.

Limit of detection (LOD) for chemical spot tests was determined by adding 200 μL of each reagent to prelabeled glass vials, followed by xylazine at concentrations of 25.0, 17.5, 10.0, 5.0, 3.75, 2.5, 1.85, 1.0, 0.5, and 0.25 mg/mL, corresponding to 5.0, 3.5, 2.0, 1.0, 0.75, 0.5, 0.37, 0.2, 0.1, and 0.05 mg of solid xylazine. Xylazine immunoassay test strips and urine dipsticks were evaluated by using solutions of 3500, 2000, 1500, 1250, 1000, 750, 500, and 0 ng/mL xylazine. For MCT, a 0.5% gold chloride aqueous reagent was tested with xylazine solutions at 1000, 750, 500, and 100 μg/mL.

Specificity testing of chemical spot tests was conducted with common xylazine adulterants, including aspirin, bis­(2,2,6,6-tetramethyl-4-piperidyl)­sebacate (BTMPS), acetaminophen, sodium bicarbonate, effervescent antacid, caffeine, and lidocaine. Mock drug mixtures combining xylazine with BTMPS, caffeine, fentanyl, and lidocaine (X, B, C, F, and L, respectively) were prepared in the following ratios: X/B, X/C, X/L, and X/F (1:1); X/F/C and X/C/L (1:1:1); and X/C/L/B (1:1:1:1). All immunoassay test strips and urine dipsticks were assessed with 1000 μg/mL of individual adulterants, including fentanyl, lidocaine, caffeine, aspirin, acetaminophen, and naproxen, and with drug cocktails (X/F/C, 1:1:1; X/C/L, 1:1:1; X/C/L/B, 1:1:1:1). Microcrystalline testing was performed using 0.5% gold chloride with adulterants (BTMPS, caffeine, levamisole, lidocaine, sodium bicarbonate) and xylazine–fentanyl mixtures in three different ratios (3:1, 1:1, 1:3).

Blind tests were conducted with xylazine and a range of drug adulterants (BTMPS, caffeine, lidocaine, and sodium bicarbonate) and over-the-counter (OTC) medications (aspirin, acetaminophen, and effervescent antacid). Each 1 mg/mL drug sample was coded alphabetically by one analyst, and a second analyst sequentially added reagents to separate vials and recorded the test results. For the immunoassay test, solutions of xylazine and drug adulterants at 1000 μg/mL were prepared and coded by one analyst, and the second analyst evaluated the performance by recording the appearance of the control and test bands. Microcrystalline testing was similarly performed using 0.5% gold chloride aqueous reagent, where individual adulterants (BTMPS, caffeine, levamisole, lidocaine, sodium bicarbonate) and mock drug mixtures of xylazine with fentanyl at different ratios were prepared and coded by one analyst. A second analyst performed the test and recorded the crystal morphology and time of crystal formation.

### 2.7 Sample Analysis and Data Processing

To enhance reproducibility and ensure the validation of results, all experiments, including chemical spot test, point-of-care, and field-deployable drug analysis, in addition to microcrystalline tests and SEM characterization, were performed in triplicate to ensure reproducibility and precision across different analysts and varying testing conditions.

## 3 Results and Discussion

### 3.1 Forensic Screening of Xylazine: Analytical Scope of Colorimetric Testing

Xylazine was evaluated using a broad range of chemical spot test reagents, including cobalt thiocyanate (CT), Dille-Koppanyi, Duquenois-Levine (DQ), Eosin Y (pH 5.0 and pH 7.0), ferric chloride, Forrest, Froehde, Janovsky, Liebermann, Mandelin, Marquis, Mecke, Nitric Acid, Scott, Simon, Young, and Zimmerman reagents. Among all of the tested reagents, no observable color change was noted with DQ, ferric chloride, Forrest, Froehde, or nitric acid, indicating negative results for these reagents in the presence of xylazine. On the other hand, Eosin Y (pH 5.0), Janovsky, Simon, and Zimmerman reagents gave faint positive results. Specifically, Eosin Y (pH 5.0) reagent showed only a slight color shift; Zimmerman reagent developed yellow specks, while Simon and Janovsky reagents produced a white precipitate upon interaction with xylazine. However, CT, Eosin Y (pH 7.0), Liebermann, Mandelin, Marquis, Mecke, Scott, and Young reagents yielded a clear and immediate color change upon the addition of xylazine, as shown in Figure 1, indicating strong positive reactions; test results are summarized in Table 1.

**Table 1:**
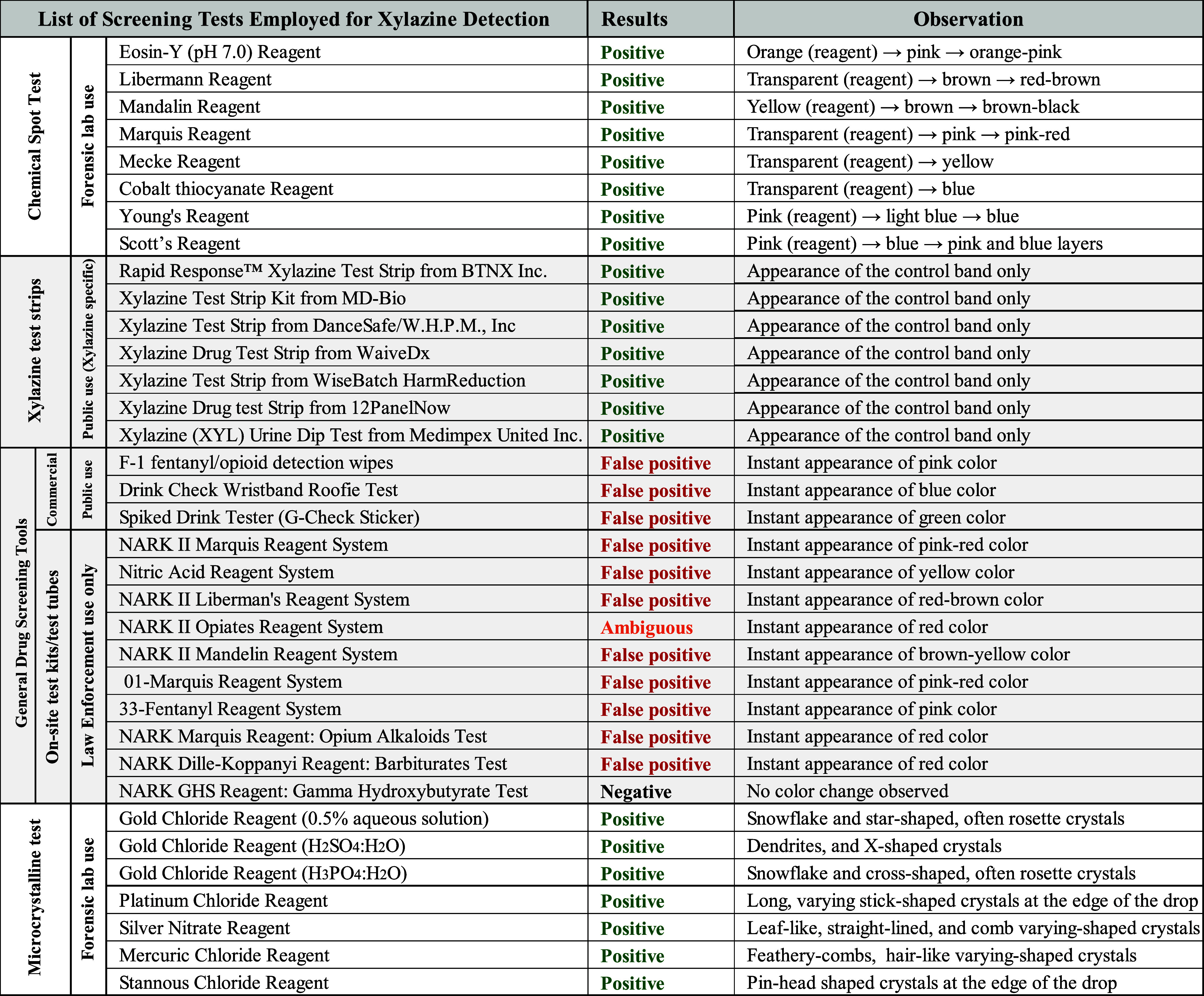
Screening Tests for Xylazine Detection, Including Chemical Spot Test, Xylazine Test Strips, Commercial and On-Site Drug Detection Tools, and a Microcrystalline Test

**Figure 1:**
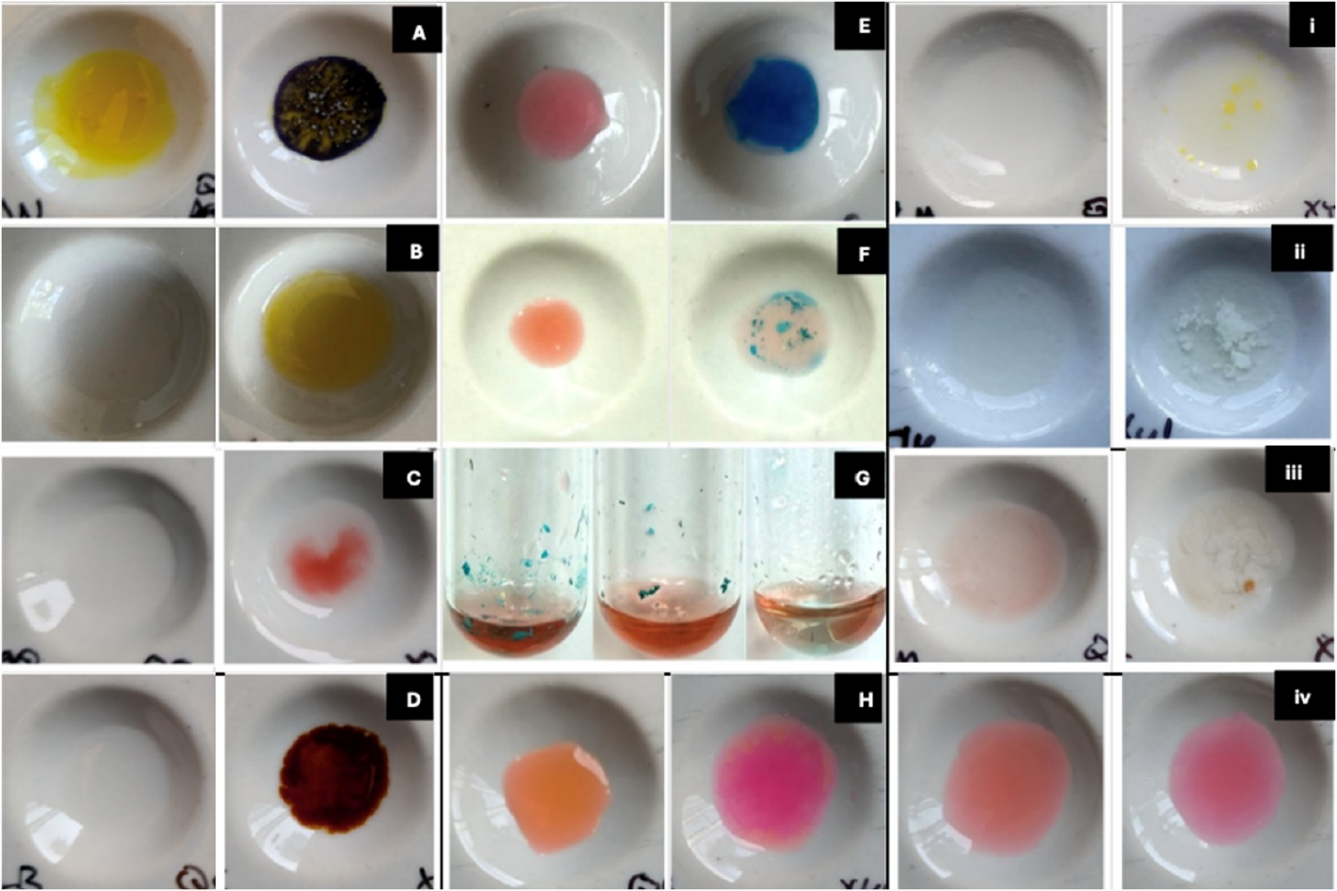
Chemical spot test of xylazine with various color test reagents. Xylazine yielded clear positive color changes with the following reagents: (A) Mandelin, (B) Mecke, (C) Marquis, (D) Liebermann, (E) cobalt thiocyanate, (F) Young, (G) Scott, and (H) Eosin Y (pH 7.0). On the other hand, reagents that produced faint or borderline positive results, characterized by minimal color change, included (i) Zimmerman, (ii) Janovsky, (iii) Simon, and (iv) Eosin Y (pH 5.0). Each pair in the color palette represents a quality control negative (reagent only) on the left side and a test mixture (reagent and xylazine) on the right side.

### 3.2 Assessment of Commercial and On-Site Drug Screening Tools

All six tested xylazine-specific immunoassay test strips and the urine dipstick yielded clear positive results, with the appearance of a single purple band at the control line (C-band) when analyzed with xylazine aqueous solution, demonstrating their effectiveness for targeted detection as illustrated in Figure 2, subset H. On the contrary, all drug screening test kits and test tubes, with the exception of the NARK GHS Reagent, which did not produce any observable color change when exposed to xylazine, demonstrated cross-reactivity, yielding false-positive results as illustrated in Figure 2, subsets A–G and I–K. The false-positive results observed in this study underscore the risk of misidentifying controlled substances when using drug screening test kits and emphasize the need for caution when law enforcement personnel employ these kits for preliminary drug identification. All drug detection tools available for general public use also yielded false-positive results in the presence of xylazine. Specifically, the drug detection wristband and the sticker responded to xylazine with a change in color from light brown to blue, erroneously indicating the presence of roofie and GHB or other sedative hypnotics. Additionally, the F-1 fentanyl/opioid detection wipes also generated a false-positive response for fentanyl when tested with xylazine. The results of xylazine test strips, point-of-care, and on-site drug screening kits are presented in Figure 2 and summarized in Table 1.

**Figure 2:**
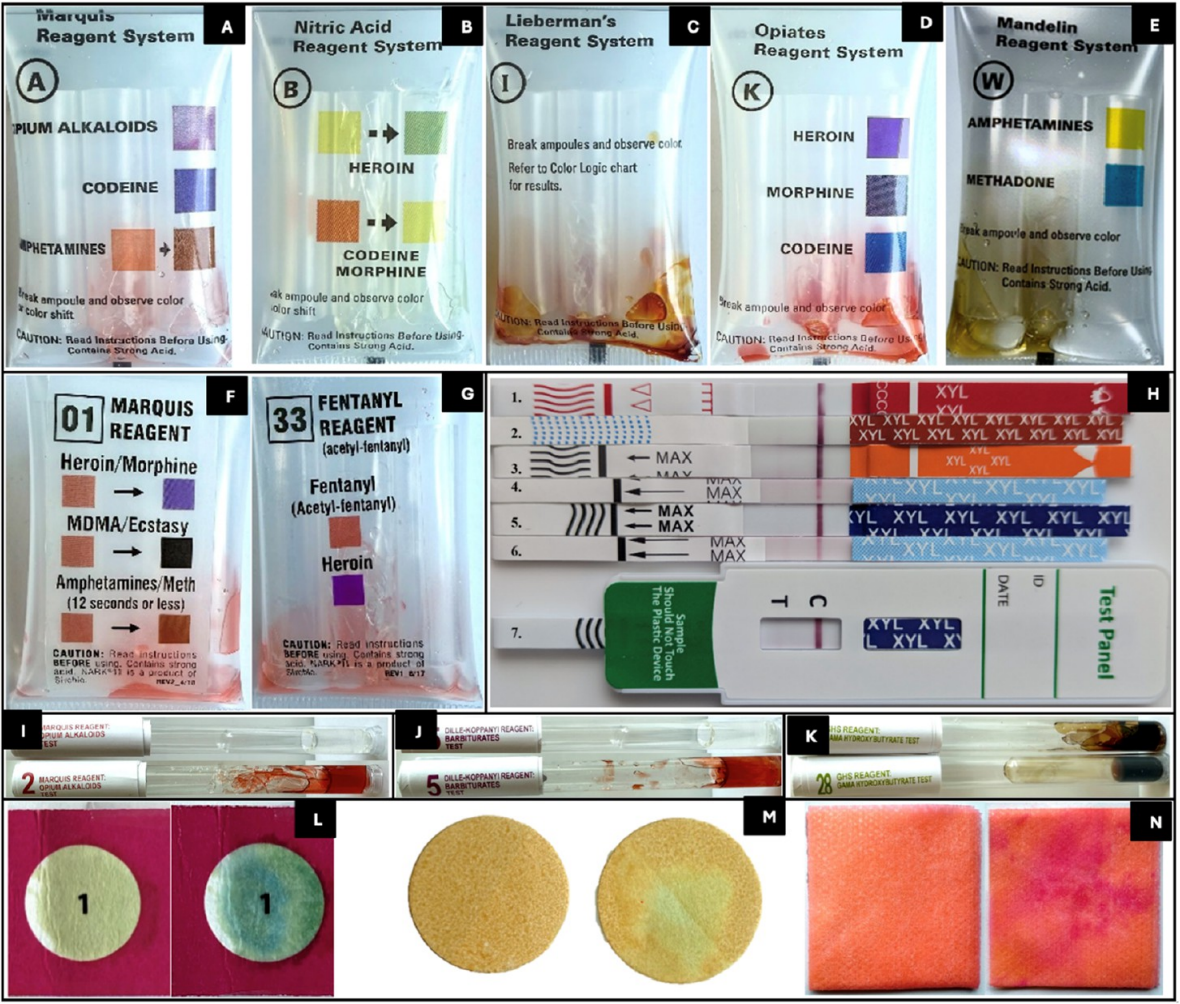
Illustration of xylazine interaction with commercial and on-site drug screening tools. (A) NARKII Marquis reagent rendered a false positive for amphetamine. (B) NARKII nitric acid reagent system yielded false positives for codeine and morphine. (C, D) NARKII Liebermann reagent system and NARKII opiate reagent system produced unique transparent-to-red-brown and red color changes, respectively. (E) NARKII Marquis reagent (opium alkaloids) yielded a false positive for amphetamine. (F) Marquis reagent gave false positives for amphetamine and methamphetamine. (G) Fentanyl reagent system yielded a false positive for fentanyl. (H) All xylazine-specific test strips and urine dipsticks yielded true-positive results when tested with xylazine solution. (I, J) NARK Marquis reagent (opium alkaloids) and NARK Dille-Koppanyi barbiturates test yielded false-positive color changes, respectively. (K) NARK GHS reagent did not produce any color change upon interaction with xylazine. (L, M) DrinkCheck Wristband Roofie Test and Spiked Drink Tester (G-Check Sticker) yielded blue color changes, giving false positives for roofie and GHB, respectively. (N) F-1 fentanyl/opioid detection wipes gave false-positive results for fentanyl.

### 3.3 Forensic Screening of Xylazine: Analytical Scope of Microcrystalline Testing

Xylazine was tested with gold chloride in three solvent systems, viz., ultrapure water, sulfuric acid (HAuCl_4_·3H_2_O in a 1:2 ratio of H_2_SO_4_ to H_2_O), and phosphoric acid (HAuCl_4_·3H_2_O in a 1:3 ratio of H_3_PO_4_ to H_2_O), following the solvent ratios described in prior studies (Figure 3, subsets G–J).^30–32^

**Figure 3:**
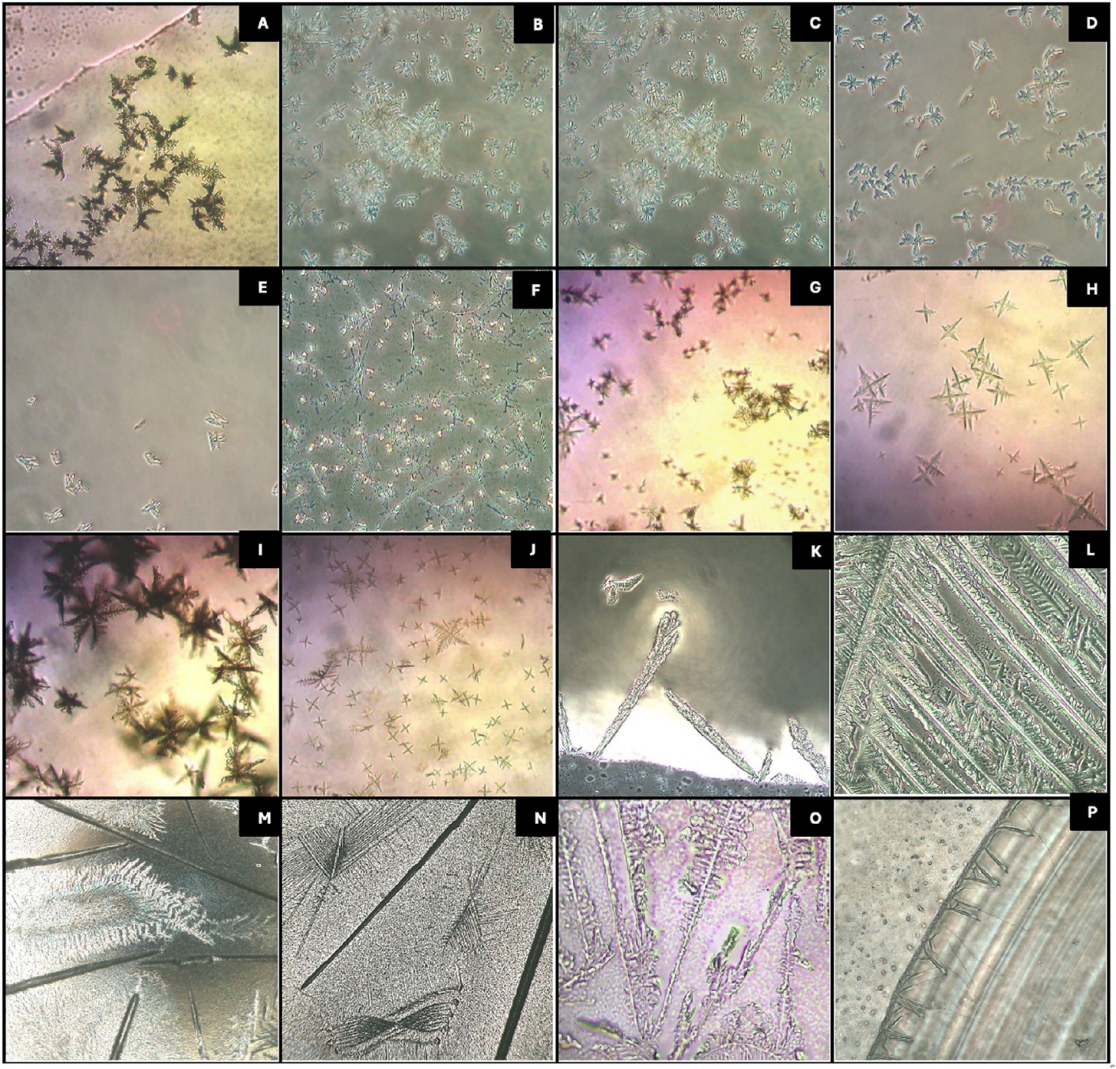
Microcrystalline test results for xylazine with various reagents. A 0.5% gold chloride aqueous reagent with (A) xylazine powder, (B–E) xylazine aqueous solutions at concentrations of 1000 μg/mL (B), 750 μg/mL (C), 500 μg/mL (D), and 100 μg/mL (E); (F) 0.5% gold chloride with 1000 μg/mL xylazine methanolic solution; (G, H) 0.5% gold chloride in sulfuric acid solvent with 1000 μg/mL xylazine aqueous (G) and methanolic (H) solutions; (I, J) 0.5% gold chloride in phosphoric acid solvent with 1000 μg/mL xylazine aqueous (I) and methanolic (J) solutions; (K) 5% platinum chloride (5%) with 1000 μg/mL xylazine aqueous solution; (L) 5% silver nitrate with 1000 μg/mL xylazine aqueous solution; (M–O) 5% mercuric chloride with xylazine powder (M), aqueous (N), and methanolic (O) solutions (1000 μg/mL); and (P) 1% stannous chloride with 1000 μg/mL xylazine aqueous solution.

Notably, xylazine yielded distinct microcrystalline formations with gold chloride reagents across all tested forms (solid, aqueous, and methanolic), independent of the solvent type. Specifically, the 0.5% aqueous gold chloride reagent produced characteristic snowflake- and star-shaped crystals, frequently forming rosettes, as consistently observed with all xylazine preparations. When gold chloride was prepared in a sulfuric acid solvent, dendritic and X-shaped crystal morphologies were observed with both aqueous and methanolic xylazine. In parallel, gold chloride in phosphoric acid yielded cross-shaped and snowflake-like crystals, often exhibiting rosette structures, again present in both aqueous and methanolic xylazine solutions. Platinum chloride yielded distinctive needle- and stick-shaped crystals, appearing primarily at the periphery of the reagent drop and only in the aqueous xylazine sample. These crystals were typically observed after a delay of several minutes. Silver nitrate produced a carpet-like arrangement of leaf-like, straight-lined, and growing, irregular blade-shaped crystals with serrated edges. However, these microcrystals were observed only upon drying and with aqueous xylazine. Mercuric chloride produced visible crystalline structures with all three xylazine forms, although crystal development required an extended time frame. The observed crystal habits included combs, feathery combs, feather-like, and straight-lined variants, but they were neither consistent nor uniform across replicates. Moreover, the negative control of mercuric chloride displayed prominent black needle-like crystals, as illustrated in Figure 3, subsets M and N. Stannous chloride yielded round-headed, pin-shaped crystals forming from a peripheral crust, visible with aqueous xylazine only after several minutes and once the drop had partially dried. Among all reagents tested, gold chloride, regardless of solvent, was the only reagent to yield immediate, reproducible, and well-defined microcrystalline structures with all forms of xylazine, making it the most reliable microcrystalline test reagent for xylazine.

### 3.4 SEM Characterization of Xylazine–Gold Chloride Crystals

Due to the immediate and reproducible results obtained with 0.5% aqueous gold chloride across all xylazine formulations, it was the only reagent selected for further analysis using scanning electron microscopy (SEM). In its solid form, xylazine reacted with 0.5% gold chloride to produce well-defined snowflake or cross-shaped crystals. The aqueous xylazine solution yielded rosette-like arrangements composed of branching serrated leaf structures, while the methanolic solution produced rosettes consisting of plate- and flower-like formations with a 0.5% aqueous gold chloride reagent, as shown in Figure 4, subsets A–C. The resulting crystal morphologies were found to be consistent, reproducible, and uniform across all three xylazine forms.

**Figure 4:**
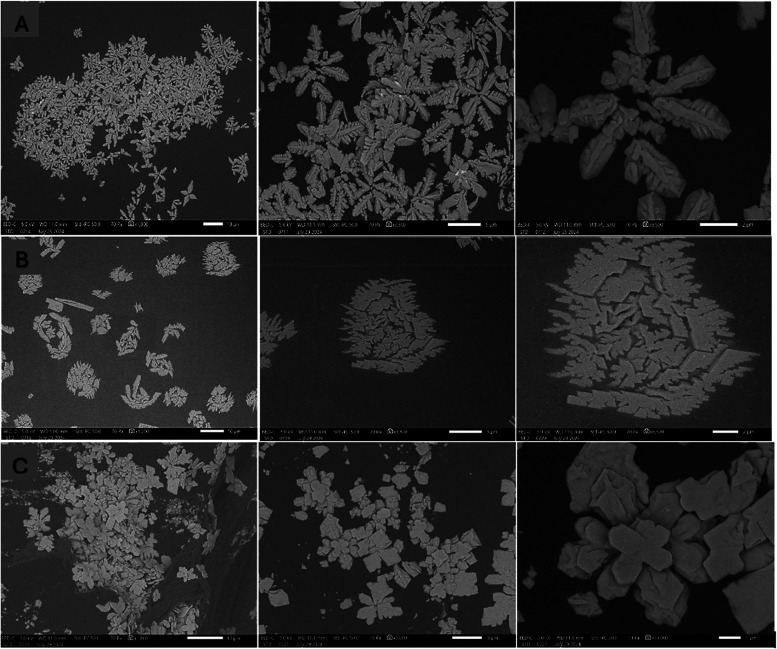
Scanning electron microscopy images of 0.5% aqueous gold chloride reagent with xylazine. Crystal morphology is shown for (A) xylazine in solid form, (B) xylazine in aqueous solution, and (C) xylazine in methanolic solution. SEM parameters for image processing and the white bar representing the scale bar are displayed at the bottom of the images. Panels (A)–(C) show images with scale bars of 10, 5, and 2 μm, respectively.

### 3.5 Performance Evaluation of Xylazine Detection Methods

#### 3.5.1 Limit of Detection (LOD)

The LOD of xylazine in chemical spot tests was determined to be 0.25 mg/mL with Liebermann, 1.0 mg/mL with Marquis, 1.85 mg/mL with Mandelin and CT, 2.5 mg/mL with Scott and Young, 3.75 mg/mL with Mecke, and 5.0 mg/mL with Eosin Y (pH 7.0), as shown in Figure 5. The Liebermann reagent demonstrated the highest sensitivity, showing a distinct color transition at the lowest xylazine concentration (0.25 mg/mL). For immunoassay-based test strips, the manufacturer-reported cutoff concentrations were 500 ng/mL for MD-Bio and 1000 ng/mL for all other test strips and the urine dipstick. However, detection thresholds higher than the manufacturer-reported cutoff concentrations were observed. Clear positive results were obtained at 2000 ng/mL for BTNx, WiseBatch, MD-Bio, WaiveDx test strips, and Medimpex urine dipstick; 1500 ng/mL for WHPM test strips; and 750 ng/mL for 12 PanelNow, as shown in Figure 5. Hence, 12 PanelNow immunoassay test strips demonstrated the highest sensitivity, yielding the lowest LOD. For microcrystalline testing, immediate and distinct snowflake-shaped crystals were observed at a minimum xylazine concentration of 500 μg/mL with 0.5% gold chloride aqueous reagent, as shown in Figure 3 (subsets B–E).

**Figure 5:**
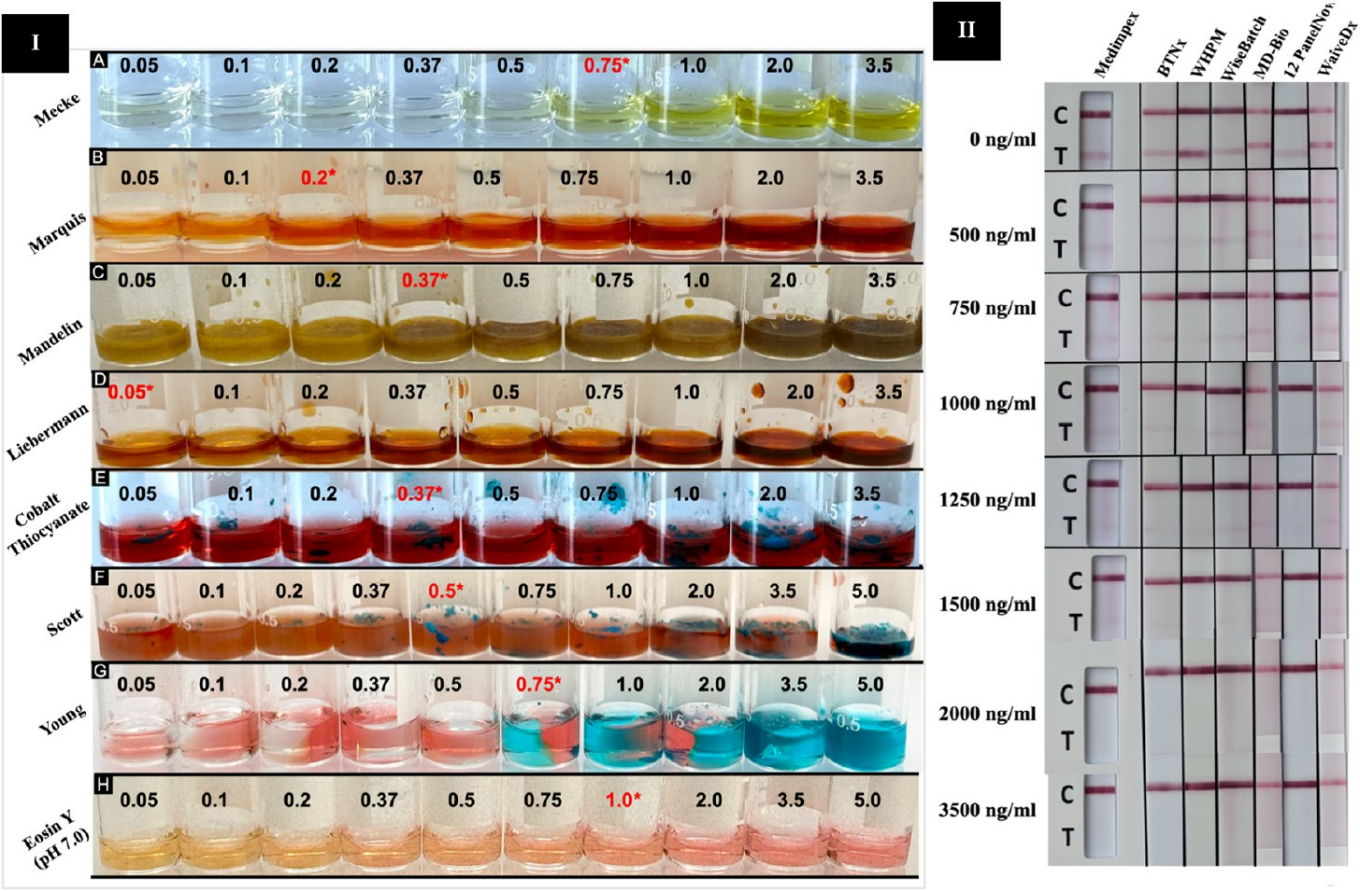
Limit of detection (LOD) study of (I) color test reagents, including (A) Mecke, (B) Marquis, (C) Mandelin, (D) Liebermann, (E) cobalt thiocyanate, (F) Scott, (G) Young, and (H) Eosin Y (pH 7.0). Each test was performed with 0.05, 0.1, 0.2, 0.37, 0.5, 0.75, 1.0, 2.0, 3.5, and 5.0 mg of xylazine. (II) LOD study of commercial xylazine immunoassay test strips and urine dipsticks at each concentration level: 0, 500, 750, 1000, 1250, 1500, 2000, and 3500 ng/mL xylazine. All tests were performed in triplicate (*n* = 3) to ensure precision and accuracy of results.

#### 3.5.2 Specificity Testing with Drug Adulterants and Mock Drug Mixtures

The specificity of xylazine detection methods was evaluated by using drug adulterants and mock drug mixtures to simulate real-world scenarios. Chemical spot test specificity experiments indicated that lidocaine yielded the same color transitions with Mandelin and Liebermann reagents as those observed with xylazine. Similarly, BTMPS yielded a response with Eosin Y (pH 7.0) comparable to that of xylazine. The analytical performance of the color tests was also influenced by drug cocktails and mock mixtures; although none reproduced the exact response of pure xylazine across all eight reagents, Mandelin, Liebermann, Scott, and Eosin Y (pH 7.0) yielded transitions most closely resembling those of xylazine for the majority of mixtures, as demonstrated in Figure 6. For the immunoassay-based tests, all drugs except lidocaine produced negative results, with both the urine dipstick and six commercial test strips. In contrast, lidocaine generated false positives with the Medimpex urine dipstick as well as the WiseBatch and 12 PanelNow test strips. All mock drug mixtures produced positive results with the immunoassay test, demonstrating that the presence of adulterants did not interfere with xylazine detection, as demonstrated in Figure 7. The microcrystalline assay exhibited high specificity for xylazine, as the crystals observed with all other drugs were morphologically distinct from the snowflake-like characteristic crystals of xylazine. However, mock mixtures of xylazine and fentanyl did not yield prominently distinct snowflake-shaped crystals, as shown in Figure 8. Future studies will focus on evaluating the effectiveness and reliability of the microcrystalline assay when xylazine is present in combination with other substances, particularly across varying compositions and ratios, to better simulate real-world scenarios encountered in seized drug casework.

**Figure 6:**
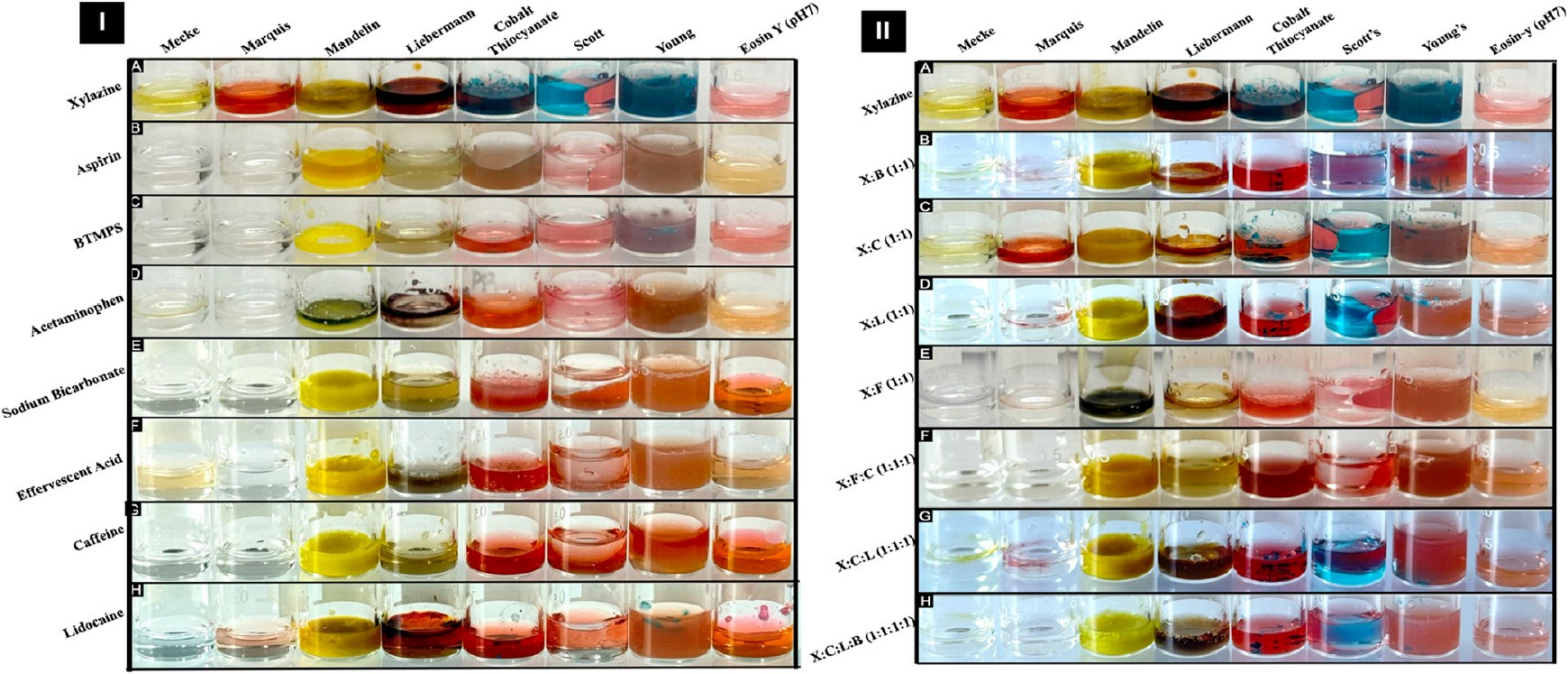
Specificity study of the color test reagents with (I) common adulterants, including (A) xylazine (positive control), (B) aspirin, (C) BTMPS, (D) acetaminophen, (E) sodium bicarbonate, (F) effervescent antacid, (G) caffeine, and (H) lidocaine, and with (II) mock drug mixtures, including (A) xylazine (positive control), (B) xylazine/BTMPS (X/B, 1:1), (C) xylazine/caffeine (X/C, 1:1), (D) xylazine/lidocaine (X/L, 1:1), (E) xylazine/fentanyl (X/F, 1:1), (F) xylazine/fentanyl/caffeine (X/F/C, 1:1:1), (G) xylazine/caffeine/lidocaine (X/C/L, 1:1:1), and (H) xylazine/caffeine/ lidocaine/BTMPS (X/C/L/B, 1:1:1:1).

**Figure 7:**
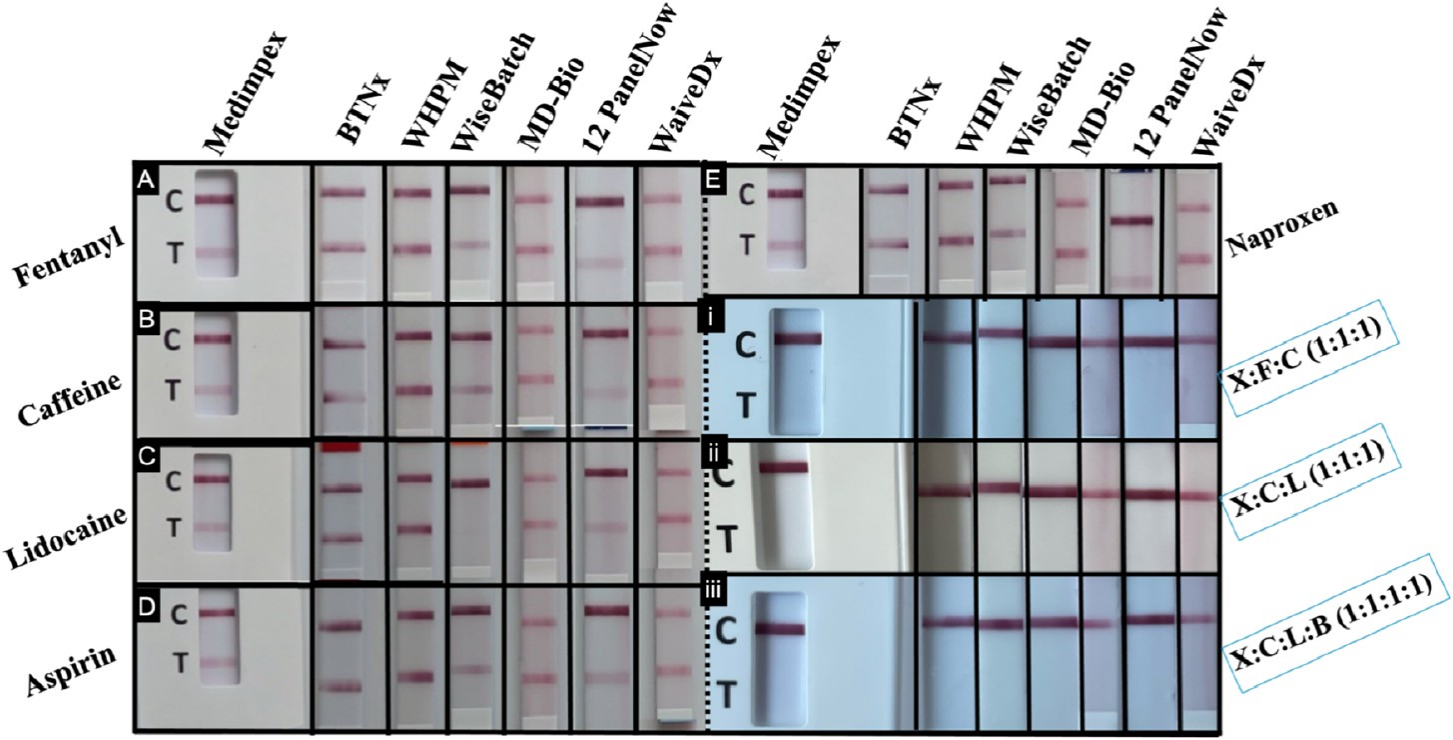
Specificity study of xylazine-specific test strips and urine dipstick with common adulterants and drug mixtures, including (A) fentanyl, (B) caffeine, (C) lidocaine, (D) aspirin, (E) naproxen, and (i) xylazine/fentanyl/caffeine (X/F/C, 1:1:1), (ii) xylazine/caffeine/lidocaine (X/C/L, 1:1:1), and (iii) xylazine/caffeine/lidocaine/BTMPS (X/C/L/B, 1:1:1:1).

**Figure 8:**
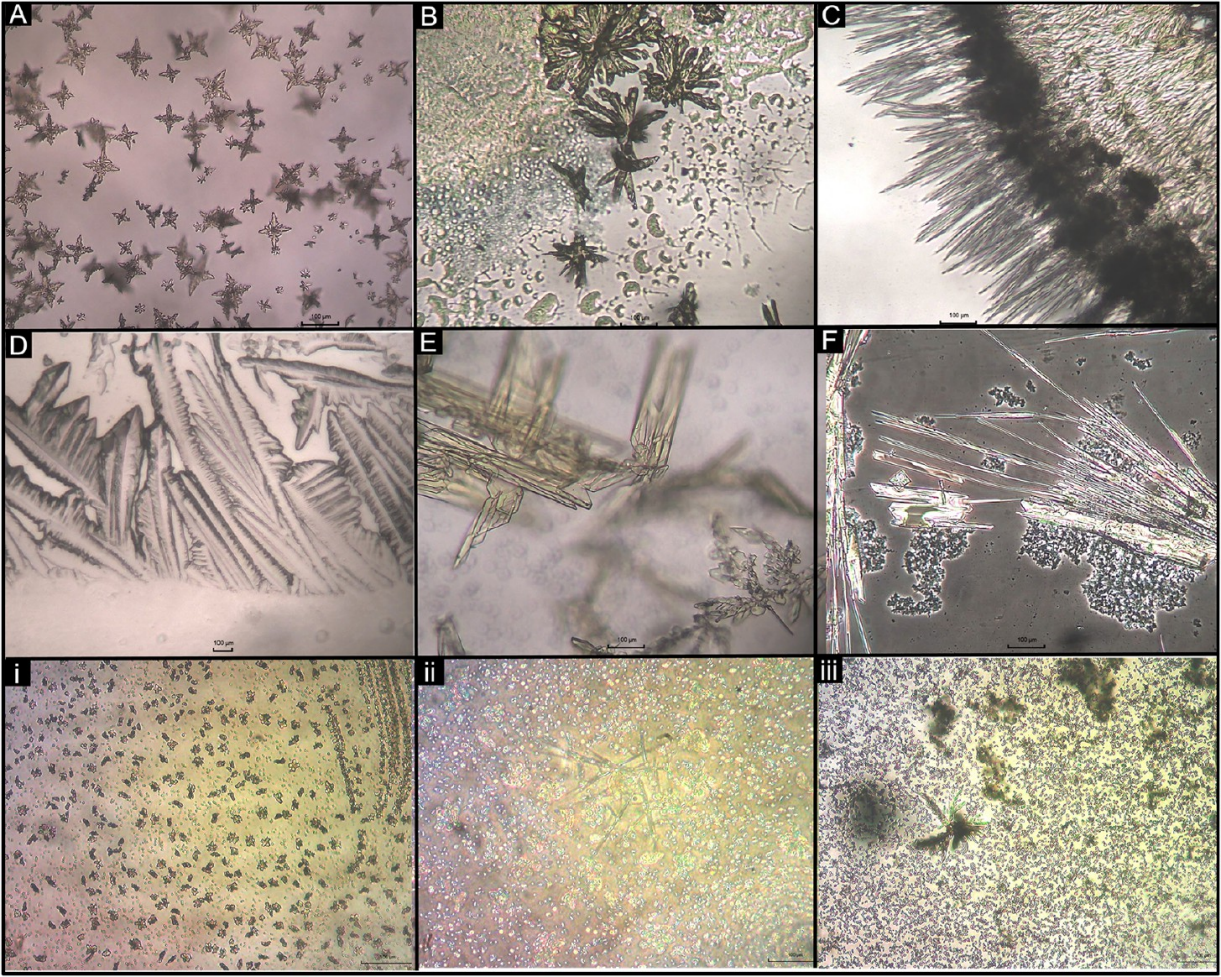
Specificity study of the microcrystalline test with 0.5% aqueous gold chloride reagent. Common drug adulterants included (A) xylazine, (B) BTMPS, (C) caffeine, (D) levamisole, (E) lidocaine, (F) sodium bicarbonate, and mock drug mixtures of xylazine and fentanyl at three ratios: (i) X/F (3:1), (ii) X/F (1:1), and (iii) X/F (1:3).

#### 3.5.3 Blind Testing

Xylazine was correctly identified in all blind trials using chemical spot tests. However, acetaminophen and lidocaine were misidentified as xylazine due to similar color transitions to the Liebermann reagent. Detection accuracy improved when at least two color test reagents were used in combination, particularly the Liebermann reagent, with one additional color test reagent. In the immunoassay tests, xylazine was correctly detected both alone and in the presence of adulterants in mock drug mixtures. Nonetheless, among the seven drugs tested individually, lidocaine produced false-positive results with the Medimpex urine dipstick as well as the WiseBatch and 12 PanelNow test strips, leading to its misidentification as xylazine. In the microcrystalline test, xylazine was consistently identified by the immediate formation of distinctive snowflake-shaped crystals, with no other compounds yielding a similar crystal morphology. However, xylazine–fentanyl mock drug mixtures did not yield distinctive snowflake microcrystals, leading to potential misidentification.

### 3.6 Results Summary and Study Limitations

To address the growing need for robust xylazine detection strategies, this study evaluated the applicability of chemical spot tests, commercial and field-deployable drug testing tools, and microcrystalline testing supported by scanning electron microscopy (SEM). Among the colorimetric reagents evaluated for their reactivity with xylazine, CT, Eosin Y (pH 7.0), and Liebermann, Mandelin, Mecke, Marquis, Scott, and Young reagents yielded rapid and distinct color changes. All xylazine-specific immunoassay test strips and urine dipsticks yielded clear positive results when tested with xylazine. In contrast, three commercially available drug detection products, viz., a drug detection wipe, wristband, and sticker (not specifically designed for xylazine), along with nine out of ten on-site drug screening kits commonly used by law enforcement, demonstrated cross-reactivity with xylazine, leading to false-positive results. It is noteworthy that several studies have reported cross-reactivity of xylazine test strips^18–20^ and false-positive results yielded by commercial and on-site drug detection kits.^33–35^ Given the significant implications of false-positive/negative results and inaccurate testing, the implementation of rigorous validation protocols for commercial and field-based testing kits is essential to safeguard public health and uphold the integrity of law enforcement efforts. In a Marijuana Policy Project report titled “False Positive Equals False Justice,” law enforcement agencies were urged to reconsider the use of unreliable field drug tests, emphasizing that the imperative to protect individuals from inaccurate results should take precedence over the convenience of flawed on-site screening tools.^36^ These findings collectively reveal the limited specificity of most of the commercial and on-site and field test kits and the urgent need to identify new xylazine-specific screening methods. It is also emphasized in this report that it is very important to conduct extensive validation of existing commercial and field drug testing tools to minimize the risk of misclassification by the public, law enforcement, and forensic personnel.^36^ Among the microcrystalline reagents tested, gold chloride was the only reagent that consistently produced immediate, well-defined, and reproducible microcrystals with all of the tested xylazine formulations, as confirmed by both light microscopy and SEM.

While this study evaluated multiple approaches for xylazine detection, it is important to acknowledge certain limitations that may affect the broader applicability of its findings. First, the scope of chemical reagents, including color spot test and microcrystalline test reagents, evaluated in this study, was restricted to those commonly used in forensic laboratories, which may exclude other reagents that could be effective for xylazine detection. Second, although the study assessed the performance of xylazine test strips and field screening tools, the results may not fully reflect their effectiveness in real-world scenarios, where factors such as the presence of adulterants, sample matrix complexity, and user variability can significantly affect the outcomes. Third, the study primarily focused on qualitative assessments, including color transition, immunoassay, and microcrystal formation with estimates of LOD and specificity for the studied methods; however, a comprehensive quantitative evaluation of the test reagents was not performed. Nonetheless, further validation with broader case studies is required to fully establish their reliability. Finally, the possibility of cross-reactivity with other substances, particularly in complex drug mixtures, was not investigated across an extensive set of simulated drug mixtures, which usually impacts the reliability of these methods in diverse casework conditions. This limits the ability to generalize the findings across broader forensic and public health settings. Future studies should address these limitations by expanding the range of reagents tested, incorporating robust quantitative analyses, and validating detection tools under field-relevant conditions, including the presence of matrix components in real-world samples, such as biological fluids or complex street drug mixtures, that may influence test performance.

## 4 Conclusions and Outlook

Given the fluctuating trends in street drug purity and the rise of designer drugs, understanding how adulterants interact with presumptive tests is critical to avoid misinterpretation, as false-positive or false-negative outcomes can compromise accuracy. This study comprehensively evaluated different testing methods for xylazine, addressing a significant gap in rapid and reliable xylazine detection tools, and presents a significant milestone in forensic chemistry. Additionally, the integration of LOD estimation, specificity assessments, and blind testing in this study highlights the need for rigorously validated detection tools for xylazine and other emerging drugs. Although immunoassay tests yielded clear positive results, a critical limitation of commercial and on-site drug screening tools that yielded false-positive results presents a serious concern because a single misinterpretation could have severe consequences and undermine the integrity of the justice system. The findings of this study also highlight the critical need for an extensive validation of the existing commercial and field drug testing tools to minimize the risk of misclassification by the public, forensic scientists, and law enforcement personnel. The findings of our study are consistent with multiple previous studies, emphasizing that the critically validated, drug-specific field tests are essential for ensuring the accuracy in frontline drug detection and safeguarding public health and justice.^18–20,33–36^ Together, these findings highlight the critical need for ongoing research, method validation, and education to ensure accurate drug detection, safeguard public health, and strengthen the integrity of forensic and harm reduction efforts.

## References

[1] Greene S. A., Thurmon J. (1988). Xylazine–a review of its pharmacology and use
in veterinary medicine. J. Vet. Pharmacol. Ther..

[2] Kariisa M., Patel P., Smith H. (2021). Notes from the field:
xylazine detection and involvement in drug overdose deaths—United
States, 2019. Morb. Mortal. Wkly. Rep..

[3] Stillwell M. E. (2003). A reported case involving impaired
driving following self-administration of xylazine. Forensic Sci. Int..

[4] Gupta R., Holtgrave D. R., Ashburn M. A. (2023). Xylazine—Medical and Public Health Imperatives. N. Engl. J. Med..

[5] Ruiz-Colon K., Martínez M. A., Silva-Torres L. A., Chavez-Arias C., Melendez-Negron M., Conte-Miller M. S., Bloom-Oquendo J. (2012). Simultaneous determination of Xylazine,
free morphine, codeine, 6-acetylmorphine, cocaine and benzoylecgonine
in postmortem blood by UPLC–MS-MS. J.
Anal. Toxicol..

[6] Friedman J., Montero F., Bourgois P., Wahbi R., Dye D., Goodman-Meza D., Shover C. (2022). Xylazine spreads across the US: a
growing component of the increasingly synthetic and polysubstance
overdose crisis. Drug Alcohol Depend..

[7] Torruella R. A. (2011). Xylazine (veterinary sedative) use
in Puerto Rico. Subst. Abuse Treat. Prev. Policy.

[8] Drug Enforcement Administration (DEA) . Growing Threat of Xylazine and its Mixture with Illicit Drugs, Oct 2022. https://www.dea.gov/sites/default/files/2022-12/The%20Growing%20Threat%20of%20Xylazine%20and%20its%20Mixture%20with%20Illicit%20Drugs.pdf (accessed Jan 2025).

[9] Office of National Drug Control Policy (ONDCP) . Fentanyl-Adulterated and or Associated with Xylazine Emerging Threat Response Plan, July 2023. https://bidenwhitehouse.archives.gov/wp-content/uploads/2023/07/fentanyl-adulterated-or-associated-with-xylazine-emerging-threat-response-plan-report-july-2023.pdf (accessed June 2025).

[10] Office of National Drug Control Policy (ONDCP) . Fentanyl Adulterated or Associated with Xylazine Implementation Report, July 2024. https://www.whitehouse.gov/wp-content/uploads/2025/03/ONDCP-2024-FAAX-Implementation-Report.pdf (accessed June 2025).

[11] Ruiz-Colón K., Chavez-Arias C., Díaz-Alcalá J. E., Martínez M. A. (2014). Xylazine
intoxication in humans and its importance as an emerging adulterant
in abused drugs: a comprehensive review of the literature. Forensic Sci. Int..

[12] Debnath R., Chawla P. A. (2023). Xylazine addiction
turning humans to zombies: Fact or myth?. Health
Sci. Rev..

[13] Center for Drug Evaluation and Research . FDA Alerts Health Care Professionals of Risks to Patients Exposed to Xylazine in Illicit Drugs, Aug 2022. https://www.fda.gov/drugs/drug-safety-and-availability/fda-alerts-health-care-professionals-risks-patients-exposed-xylazine-illicit-drugs (accessed May 2025).

[14] Alapati A., Nugent K. (2025). Xylazine toxicity. Southwest J. Med..

[15] Mittleman R. E., Hearn W. L., Hime G. W. (1998). Xylazine
toxicity-literature review and report of two cases. J. Forensic Sci..

[16] Carruthers S. G., Nelson M., Wexler H., Stiller C. (1979). Xylazine hydrochloridine
(Rompun) overdose in man. Clin. Toxicol..

[17] Chang H. Y., Donnachie K., McCabe T. J., Barrington H., Carlysle-Davies F., Ceniccola-Campos K., Reid M. (2025). Presumptive Tests for
Xylazine—A Computer Vision Approach. Anal. Sci. Adv..

[18] Sisco E., Nestadt D. F., Bloom M. B., Schneider K. E., Elkasabany R. A., Rouhani S., Sherman S. G. (2024). Understanding sensitivity
and cross-reactivity of xylazine lateral flow immunoassay test strips
for drug checking applications. Drug Test. Anal..

[19] British Columbia Centre on Substance Use . Detection of Xylazine by Immunoassay Test Strips in Community Drug Samples: A Preliminary Report, May 2024. https://drugcheckingbc.ca/wp-content/uploads/sites/4/2024/05/2024-04-26-Detection-of-xylazine-by-immunoassay-test-strips-in-community-drug-samples-A-preliminary-report-FINAL-May-2024.pdf (accessed Jan 2025).

[20] Thompson E., Tardif J., Ujeneza M., Badea A., Green T. C., McKee H., McKenzie M., Park J. N. (2024). Pilot findings on the real-world performance of xylazine
test strips for drug residue testing and the importance of secondary
testing methods. Drug Alcohol Depend. Rep..

[21] Scott L., Davis K., Park J. N., Majeed S. (2025). Evaluating the Sensitivity, Selectivity, and Cross-Reactivity
of Lateral Flow Immunoassay Xylazine Test Strips. J. Appl. Lab. Med..

[22] Philp M., Shimmon R., Stojanovska N., Tahtouh M., Fu S. (2013). Development and validation of a presumptive
colour spot test method for the detection of piperazine analogues
in seized illicit materials. Anal. Methods.

[23] Philp M., Fu S. (2018). A review of chemical
‘spot’tests: A presumptive illicit drug identification
technique. Drug Test. Anal..

[24] Harper L., Powell J., Pijl E. M. (2017). An overview
of forensic drug testing methods and their suitability for harm reduction
point-of-care services. Harm Reduct. J..

[25] O’Neal C. L., Crouch D. J., Fatah A. A. (2000). Validation of twelve
chemical spot tests for the detection of drugs of abuse. Forensic Sci. Int..

[26] Cabeza A. S., Falcó P. C., Legua C. M. (1994). Kinetic-Spectrophotometric Determination of Primary
and Secondary Amines by Reaction with 1–2 Naphthoquinone-4-Sulphonate. Anal. Lett..

[27] Moffat, A. C. ; Osselton, M. D. ; Widdop, B. ; Watts, J. Clarke’s Analysis of Drugs and Poisons, 3rd ed.; Pharmaceutical Press: London, 2011; p 2535.

[28] Elie, M. P. ; Elie, L. E. Microcrystalline Tests in Forensic Drug Analysis. In Encyclopedia of Analytical Chemistry; Wiley, 2007; Vol. 1, pp 471–481.

[29] Elie L. E., Baron M. G., Croxton R. S., Elie M. P. (2011). Reversing microcrystalline
tests—An analytical approach to recycling of microcrystals
from drugs of abuse. Forensic Sci. Int..

[30] Brinsko, K. M. ; Golemis, D. ; King, M. B. ; Laughlin, G. J. ; Sparenga, S. B. A Modern Compendium of Microcrystal Tests for Illicit Drugs and Diverted Pharmaceuticals, Compendium, 2016.

[31] Quinn, M. ; Cain, M., Jr ; Joshi, M. Library of Microcrystalline Tests for Novel Psychoactive Substances, 2017.

[32] Brinsko, K. M. ; Golemis, D. ; King, M. B. ; Laughlin, G. J. ; Sparenga, S. B. A Modern Compendium of Microcrystal Tests for Illicit Drugs and Diverted Pharmaceuticals. McCrone Research Institute: Chicago, 2015.

[33] Cosby, D. A. Adulterants and Interpretive Challenges in Forensic Science: Effects on Colorimetric Spot Tests for Presumptive Drug Identification and Adverse Side Effects in the Body. PhD Thesis, Boston University, 2014.

[34] Kosecki P.
A., Brooke P., Canonico E. (2022). Fentanyl as a potential false positive with color tests
commonly used for presumptive cocaine identification. J. Forensic Sci..

[35] Grates, K. M. ; Ring, J. G. ; Savage, K. A. ; Denicola, T. A. ; Beall, V. ; Dovyak, J. ; Schlomer, E. Conclusion of Validation Study of Commercially Available Field Test Kits for Common Drugs of Abuse; NFSTC: USA, 2008.

[36] Kelly, J. Marijuana Policy Project. False Positive Equals False Justice, 2008. https://www.mpp.org/files/uploads/2016/01/falsepositives.pdf#page=25.13 (accessed June 2025).

